# Revisiting the cognitive advantages of professional soccer players

**DOI:** 10.1073/pnas.2515523123

**Published:** 2026-02-18

**Authors:** Jack Fitzgerald, Niklas Jakobsson, Abel Brodeur

**Affiliations:** ^a^Department of Ethics, Governance, and Society, Vrije Universiteit Amsterdam, Amsterdam 1081 HV, The Netherlands; ^b^Tinbergen Institute, Amsterdam 1082MS, The Netherlands; ^c^Karlstad Business School, Karlstad University, Karlstad 651 88, Sweden; ^d^Department of Economics University of Ottawa, Ottawa, ON K1N 6N5, Canada; ^e^Institute for Replication, University of Ottawa, Ottawa, ON K1N 6N5, Canada

Bonetti et al. (1) analyzes cognitive data on 204 professional soccer players from Brazil and Sweden and 124 nonelite Brazilian controls, reporting that elite players score detectably higher than controls on several cognitive measures. We document two issues.

First, these results are potentially driven by poor sample selection. The average Brazilian control participant in this study is 27.38 y old and has 8.01 y of formal education (pgs. 7–8). This is over 2 y less than the 10.1 y of education attained by the average Brazilian aged ≥ 25 y (2). These controls are selected to “match” professional players’ average of 8.74 y in formal education (pg. 7). However, elite players likely exit education early not because of academic difficulty, but rather due to conflicts with training in football academies. In contrast, though not the only reason why, one reason why the study’s controls may have exited formal schooling early is due to experiencing more difficulty with school. This confounding could induce differences in cognitive scores between the professional athletes and controls in the study’s sample that are unrelated to soccer skill.

The Swedish subset of the paper’s replication data (3) permits better comparisons. All 51 Swedish players are professional, but some are of such high quality that they were selected for national teams. These players’ scores on the Trail Making Test, Design Fluency Test, and Color-Word Interference Test enable cognitive comparisons between national and just-professional players. Some of the paper’s authors make similar comparisons in prior work, from which the Swedish data is copied (4).

**Fig. 1.**
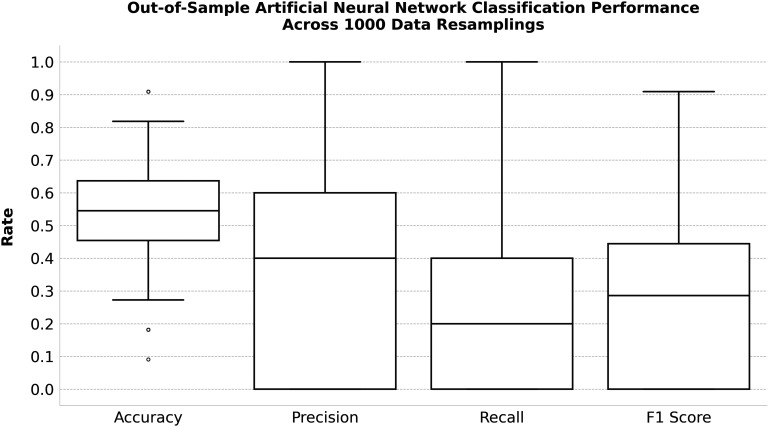
Out-of-sample performance is evaluated on 1,000 resamplings of the Swedish data. For each resampling, the 51 Swedish players are randomly assigned to training and testing data with 80% and 20% probability (respectively). Estimates arise from comparing predicted classifications with true classifications for players assigned to the testing data. Classification predictions are generated by an adapted version of the artificial neural network models applied in Soccer_ArtificialNeuralNetworks_v2.py from the paper’s replication repository (3), using all available cognitive scores in the replication data as features. Box and whisker plots show the distributions of each performance metric, displaying the 25th, 50th, and 75th percentiles of a given metric in the box, the range of all observed values of that metric within 1.5 interquartile ranges of the box in the whiskers, and all observed values further than 1.5 interquartile ranges away from the box displayed as individual outlier points.

The paper’s results do not replicate in the Swedish data. Our Table 1 shows that most differences in cognitive scores between national and just-professional players are not robustly statistically significantly different from zero. Though the lack of statistically significant differences can be partly explained by a loss of power, our Fig. 1 additionally shows that cognitive scores offer no predictive power. The same artificial neural networks reported to distinguish Brazilian professional players from poorly matched controls with 96.9% average accuracy could only distinguish Swedish national players from just-professional players with 53% average accuracy, near the no-information rate of 54.9%.

**Table 1.** Average Differences in Cognitive Scores Between National and Just-Professional Players

Test	Coefficient	SE	*P*-value	Bonferroni–Holm *q*-value	DF
TMT-1	0.351	0.472	0.461	0.930	49
TMT-2	−1.099	0.643	0.093	0.566	49
TMT-3	−0.638	0.432	0.146	0.731	49
DF-1	−0.703	0.764	0.362	0.930	49
DF-2	−0.576	0.561	0.310	0.930	49
DF-3	−2.04	0.84	0.019	0.151	49
CWI-1	−0.64	0.467	0.177	0.731	49
CWI-2	−1.643	0.516	0.003	0.025	49
CWI-3	−1.238	0.694	0.081	0.566	49
CWI-4	−1.411	0.565	0.016	0.142	49

*Note*: Coefficients arise from simple ordinary least squares models that regress the cognitive score designated by the row on a dummy variable indicating whether a player is a just-professional player rather than a national player (4). TMT, DF, and CWI respectively correspond to scales of the Trail Making Test, the Design Fluency Test, and the Color-Word Interference Test. *P*-values are computed using HC3 heteroskedasticity-robust SEs (5). Bonferroni–Holm *q*-values are computed to control family-wise error rates over all ten tests reported in Table 1. DF indicates residual degrees of freedom. Data are restricted to the 51 Swedish players in the paper’s replication data (3). Note that the test results in the original paper’s Table 1 for TMT-1, TMT-2, and TMT-3, respectively, arise from scores labeled “TMT2scale,” “TMT3scale,” and “TMT4scale” in the paper’s replication data; we retain the paper’s notation for consistency.

Second, some of the paper’s statistical evidence is mischaracterized. Table 1 is introduced as presenting *two*-sample t-tests between professional players and comparison groups (pg. 3). However, the replication repository’s R code (5) calls t.test() with a single score vector and argument mu = 10. This syntax executes *one*-sample *t* tests against a fixed mean of 10, not two-sample tests of differences between groups. Additionally, there is no documentation justifying that this mean of 10 is a representative average of cognitive scores for the specific countries and demographic profiles represented in the paper’s data. A correction to the original article has since been posted concerning this matter (6), which was originally identified by our author team.

Taken together, these issues suggest that one of the paper’s central claims—that elite soccer players exhibit detectable cognitive advantages—is not reliably demonstrated. Replication data and code for this paper is available at ref. 7.
